# Supplementary material for: Transition to naïve human pluripotency mirrors pan-cancer DNA hypermethylation
Source: Nat Commun. 2020 Jul 22;11:3671. doi: 10.1038/s41467-020-17269-3 (PMC7376100; doi:10.1038/s41467-020-17269-3)
Supplement: Supplementary file 1 — Supplementary Information [file 41467_2020_17269_MOESM1_ESM.pdf]

Supplementary Information

Transition to naïve human pluripotency mirrors pan-cancer DNA hypermethylation

Patani H et al.

Supplementary Figure 1: DNA methylation changes during primed to naïve resetting

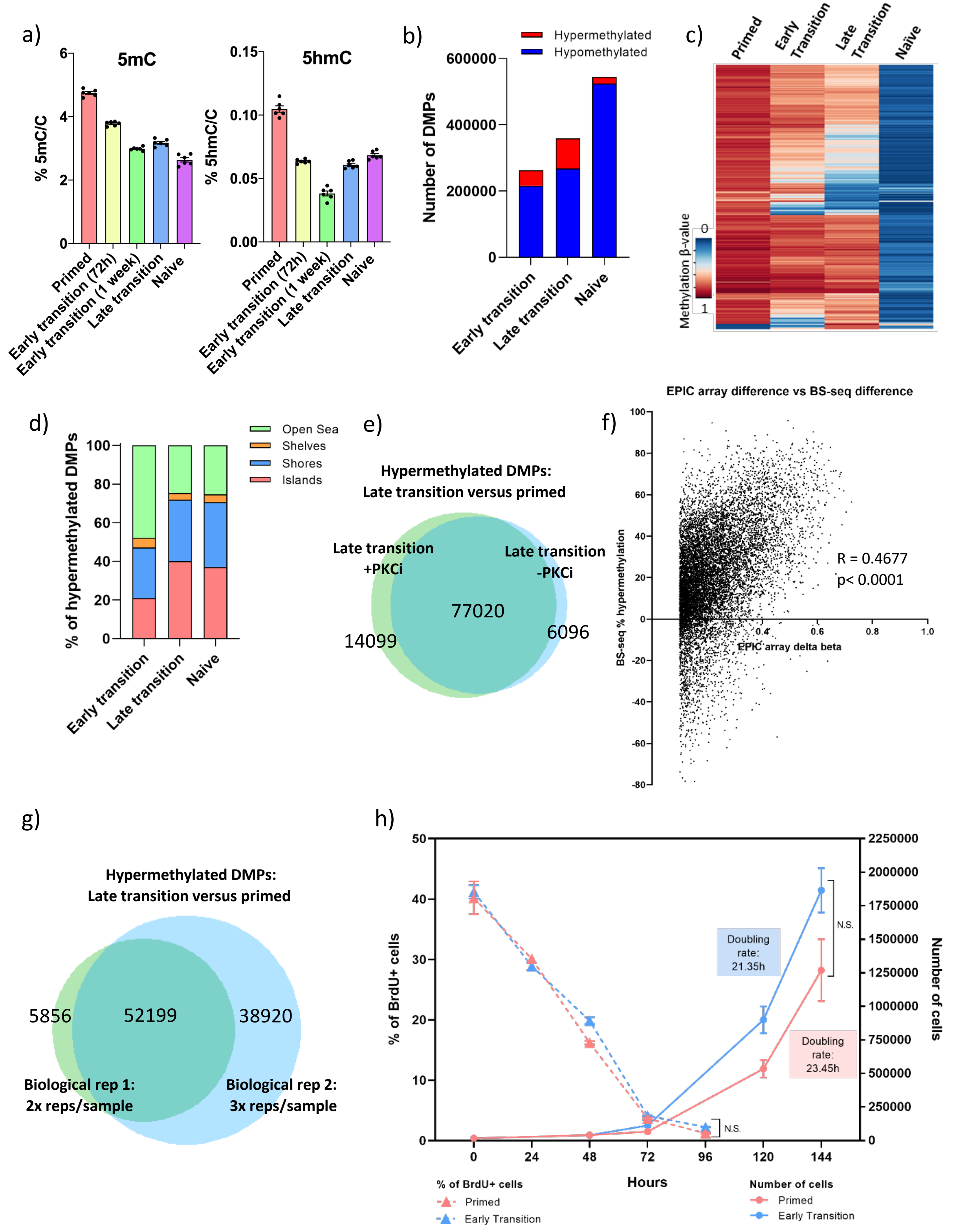

Supplementary Figure 1. DNA methylation changes during primed to naïve resetting. a) Mass spectrometry analysis of 5-methylcytosine (5mC) and 5-hydroxymethylcytosine (5hmC) at the time points analysed. Bars represent the mean of two biological replicates each with three technical replicates. b) Differentially methylated probes (DMP) identified from the Infinium Methylation EPIC array as either hypomethylated ($\Delta\beta < 0.1$, adjPval < 0.05) or hypermethylated ($\Delta\beta > 0.1$, adjPval < 0.05) at each of the time points compared to primed hESCs. AdjPval is based on Benjamini-Hochberg adjustment. c) Heatmap showing methylation levels of the top 20,000 CpG probes that are hypomethylated ($\Delta\beta < 0.1$, adjPval < 0.05) in the early transition, late transition and naïve hESCs compared to primed hESCs. Methylation β-value is indicated by the colour key. adjPval based on Benjamini-Hochberg adjustment. d) Proportion of hypermethylated probes ($\Delta\beta > 0.1$, adjPval < 0.05) at each time point compared to primed hESCs that fall within CpG islands, shores, shelves and open sea. e) Venn diagram showing the overlap of hypermethylated DMP between the late transition and primed hESCs in the presence or absence of PKCi (Gö), indicating that DNA hypermethylation is not dependent on PKCi. f) Scatter plot showing the correlation between the difference in methylation (delta beta) between naïve and primed hESCs for hypermethylated probes from the EPIC array and the % hypermethylation from published Takashima WGBS data for 100bp probes centered over the EPIC array probes. A two-sided Pearson's correlation is used to calculate correlation between the data sets. g) Venn diagram showing the overlap of hypermethylated DMP between the late transition and primed hESCs between two independent Infinium Methylation EPIC arrays. h) Cell population doubling for primed and early transition hESCs (right y-axis) and the rate of loss of BrdU over time for primed and early transition hESCs that begin with equal levels of DNA-incorporated BrdU on day 0. Statistical differences between samples were calculated using a two-tailed paired t-test. N.S. denotes not significant. Source data are provided as a Source Data file.

Supplementary Figure 2: Overlap of WGBS (Takashima) hypermethylated regions with histone modifications and regulatory elements

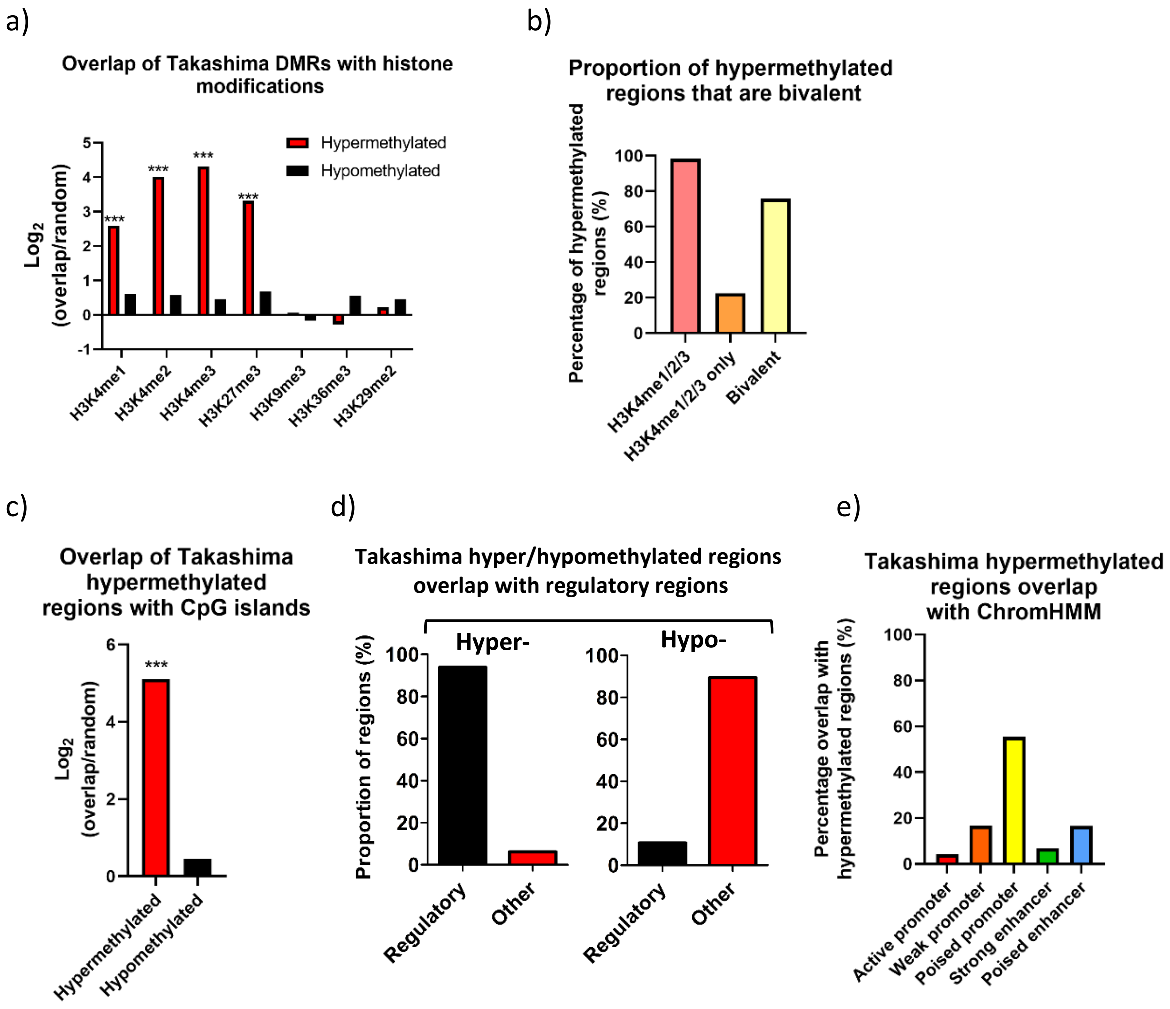

Supplementary Figure 2. Overlap of WGBS (Takashima) hypermethylated regions with histone modifications and regulatory elements. a) Overlap of 300bp hypermethylated regions (>10% methylation in naïve vs primed hESC, n = 18,675) and hypomethylated regions (<10% methylation in naïve vs primed hESC, n = 1,772,933) identified from published (Takashima) WGBS data with regions of histone modification enrichment (obtained from the ENCODE ChIP-seq data for hESC cell line H1: H3K4me1 n = 139971; H3K4me2 n = 73086; H3K4me3 n = 33270; H3K9me3 n = 86122; H3K27me3 n = 25909; H3K36me3 n = 35877; H3K79me2 n = 33205). b) The proportion of hypermethylated regions (n = 18,675) that overlap H3K4me1/2/3 or H3K27me3 or bivalent regions (marked by H3K4me3 and H3K27me3). c) Overlap of Takashima hypermethylated and hypomethylated regions (as in 2a) with CpG islands (n = 30344). Data is presented as the $\log_2$ corrected fold increase in the observed overlap compared to the mean overlap of 1000 randomly generated regions. d) Overlap of Takashima hypermethylated and hypomethylated regions (as in 2a) with ENCODE regulatory regions (promoters and enhancers as defined in the H1 hESC cell line). e) Proportion of Takashima hypermethylated regions (n = 18,675) that overlap with ENCODE predicted promoters and enhancers (as defined by ChromHMM in the hESC cell line H1).

For the overlap analysis, data is presented as the $\log_2$ corrected fold increase in the observed overlap compared to the mean overlap of 1000 randomly generated regions. ***P< 0.001.

Supplementary Figure 3. Hypermethylation is a hallmark of the transition to naïve human pluripotency

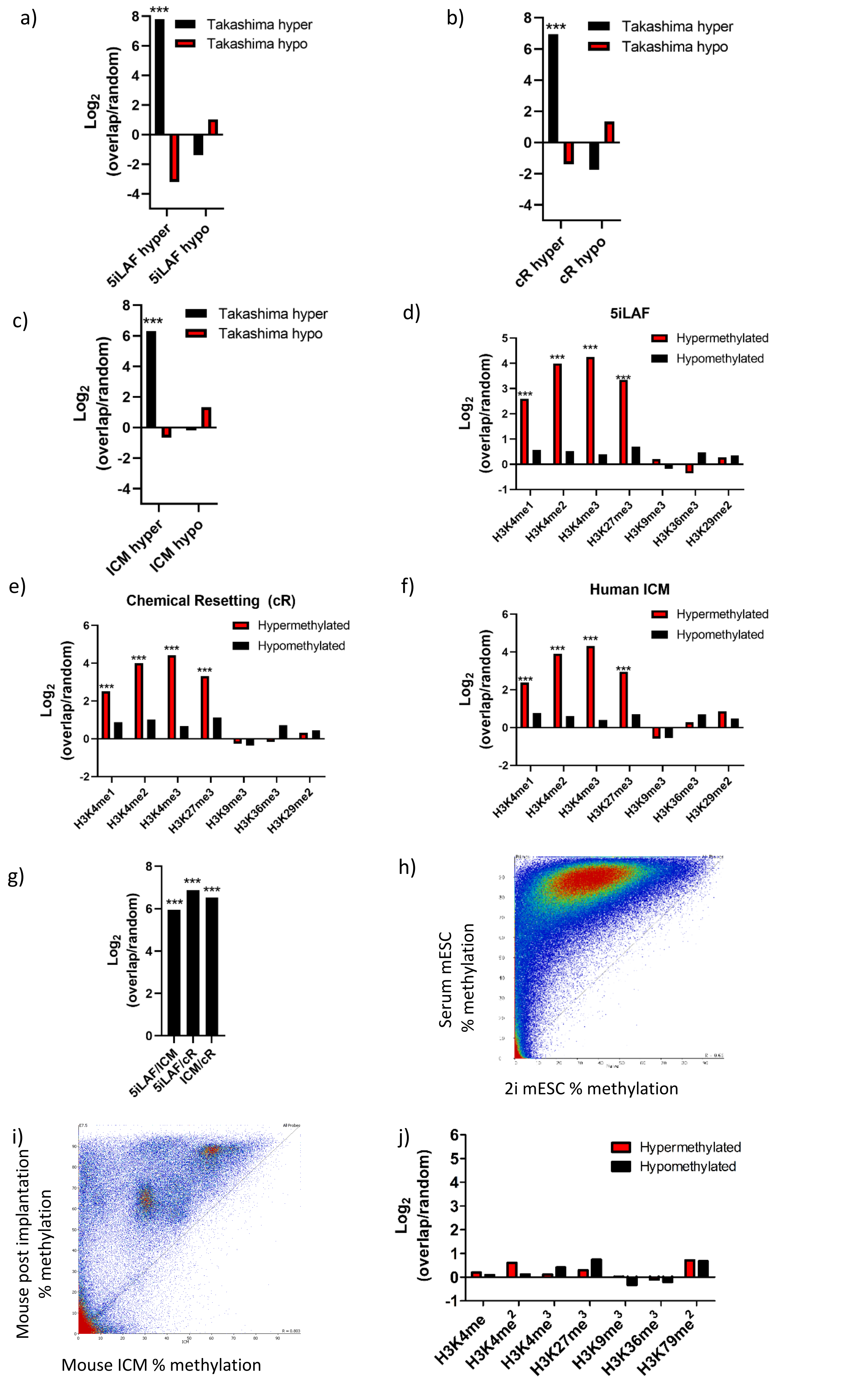

Supplementary Figure 3. Hypermethylation is a hallmark of the transition to naïve pluripotency.
a) Overlap of 300bp hypermethylated (n = 18,675) and hypomethylated regions (n = 1,772,933) identified from published WGBS data with 300bp naïve hypermethylated and hypomethylated regions identified from published data for the 5iLAF resetting method (n = 8,392 hypermethylated, n = 2,315,321 hypomethylated). b) Overlap of 300bp Takashima naïve hypermethylated and hypomethylated regions (as in 3a) with 300bp naïve hypermethylated (n = 26,090) and hypomethylated regions (n = 541,648) identified from published data for the chemical resetting method. c) Overlap of 300bp Takashima naïve hypermethylated and hypomethylated regions (as in 3a) with 300bp regions identified as hypermethylated (n = 6413) or hypomethylated (n = 104,471) in human ICM compared to post-implantation embryo from published data. d) Overlap of 300bp 5iLAF naïve hypermethylated and hypomethylated regions (as in 3a) with ENCODE histone modifications (H3K4me1 n = 139971; H3K4me2 n = 73086; H3K4me3 n = 33270; H3K9me3 n = 86122; H3K27me3 n = 25909; H3K36me3 n = 35877; H3K79me2 n = 33205) . e) Overlap of 300bp chemically reset naïve hypermethylated and hypomethylated regions (as in 3b) with ENCODE histone modifications (as in 3d). f) Overlap of 300bp ICM hypermethylated and hypomethylated regions (as in 3c) with ENCODE histone modifications (as in 3d). g) Overlap of 300bp hypermethylated regions identified from published data for the 5iLAF resetting method, the chemical resetting (cR) method and the human ICM with each other (as in 3a, 3b, 3c). h) Scatter plot showing methylation % for 300bp fragments of DNA in mouse ESCs cultured in serum or 2i. i) Scatter plot showing methylation % for 300bp fragments of DNA in mouse ICM vs post-implantation epiblast. j) Overlap of 300bp mouse ICM hypermethylated regions with ENCODE histone modifications.
For overlap analysis, data is presented as the $\log_2$ corrected fold increase in the observed overlap compared to the mean overlap of 1000 randomly generated regions. ***P< 0.001.

Supplementary Figure 4: Gene ontology of hypomethylated and hypermethylated genes

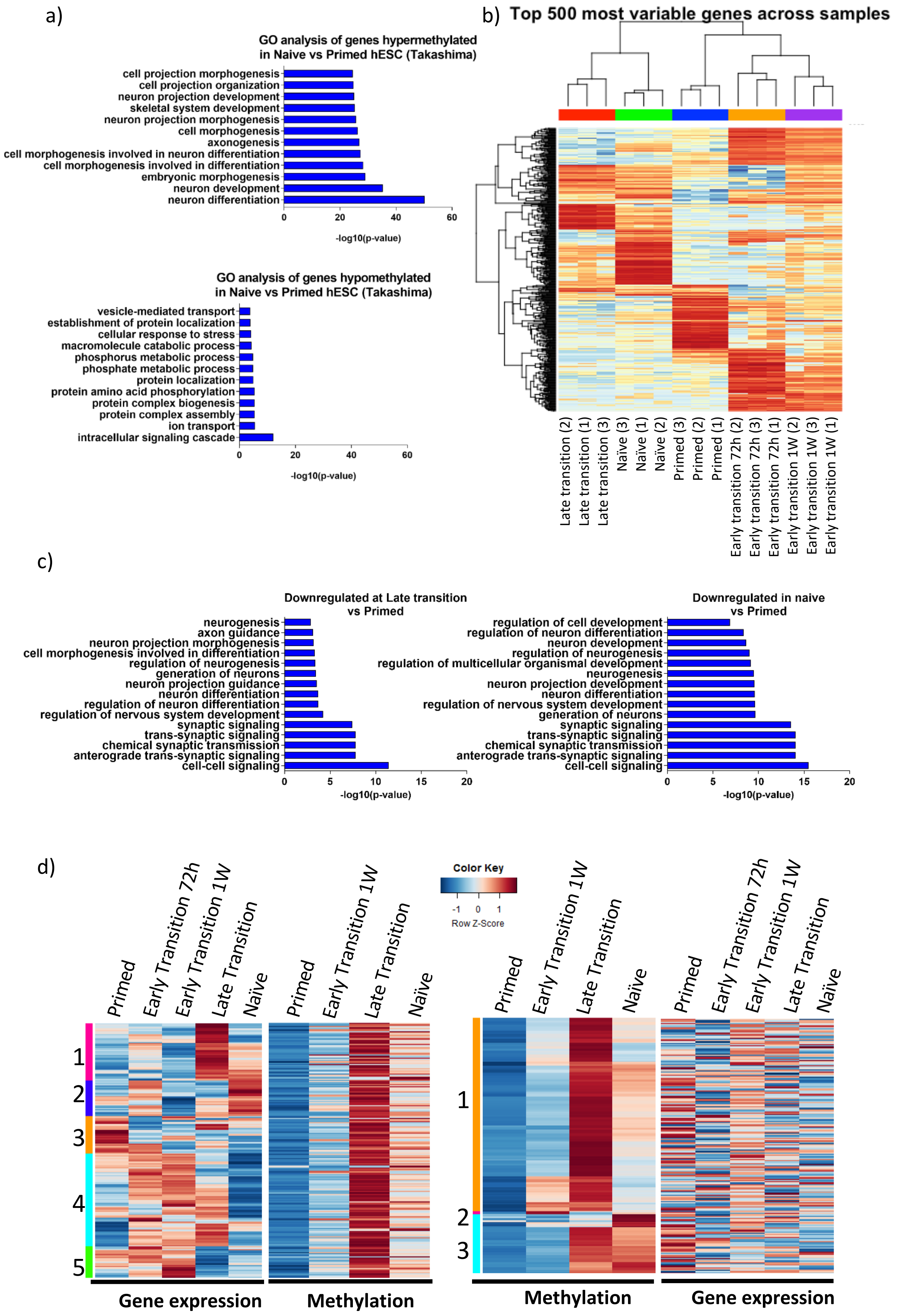

Supplementary Figure 4. Gene ontology of hypomethylated, hypermethylated and downregulated genes a) GO term analysis of hypermethylated and hypomethylated genes in naïve hESCs compared to primed hESCs. A gene was classified as hypermethylated based on the closest overlapping gene to the 300bp regions identified as hypermethylated from WGBS published data[1]. b) MDS plot show the top 500 most variably expressed genes from RNA-seq data. Replicates for each time point cluster together. c) GO term analysis of genes downregulated (log2 fold change > 2, $p < 0.05$) at the late transition of resetting or in naïve hESCs compared to primed hESCs. d) Heatmaps showing clustered gene expression data (logCPM) or clustered DNA methylation (beta-values of average promoter methylation) data for genes that are hypermethylated in naïve compared to primed hESCs (average promoter methylation $\Delta\beta > 0.1$ in naïve vs primed hESC) with the associated DNA methylation dynamics or gene expression dynamics displayed on the heatmap alongside. Z-scores for scaled data on each heatmap is indicated by the colour key.

Supplementary Figure 5: Dynamics of DNA methylation regulators during resetting

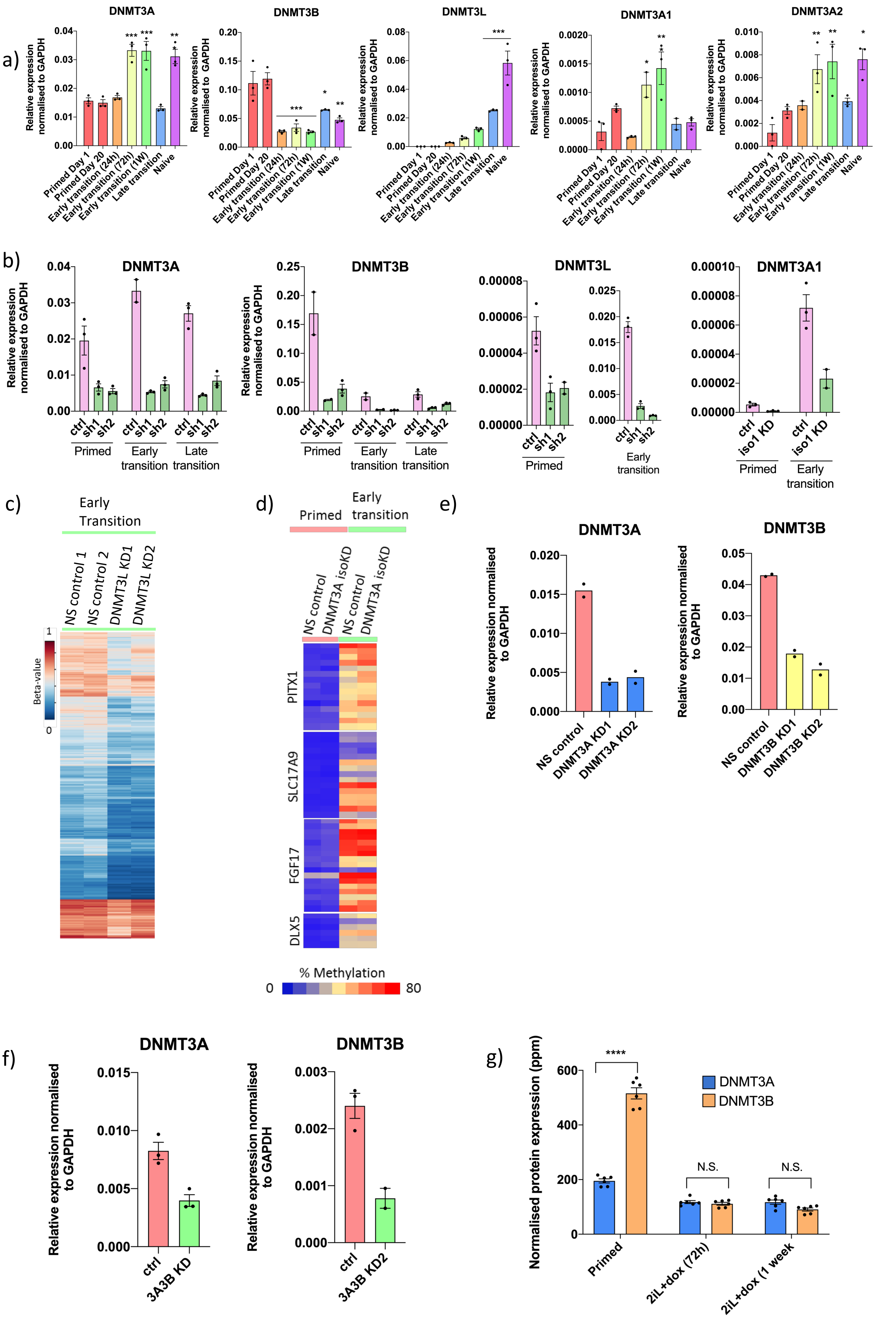

Supplementary Figure 5. Dynamics of DNA methylation regulators during resetting. a) qRT-PCR for the transcripts of the DNMT3 family across the period of resetting. Bars are representative of three independent biological replicates with two technical replicates. Error bars represent the standard error of the mean. Statistical difference between samples was analysed by a one way ANOVA test. *$P<0.05$, **$P<0.01$, and ***$P<0.0001$. Human GAPDH was used to normalise expression. b) qRT-PCR for the transcripts of the DNMT3 family in control and knock-down cell lines, across the period of resetting. Bars represent the mean of three technical replicates and error bars represent the SEM. Human GAPDH was used to normalise expression. c) Heatmap showing methylation levels for early transition control and DNMT3L knock down samples. Heatmap is composed of the top 17,000 CpG differentially methylated probes (DMP; $\Delta\beta > 0.1$, $p < 0.05$) in the early transition compared to primed hESCs (in wild type early transition compared to primed hESCs identified in analysis shown in Fig 1b). Methylation β-value is indicated by the colour key. d) Targeted bisulfite-sequencing of 4 regions of DNA. Each square represents the methylation % indicated by the colour key of a single CpG. Data is shown for a knock down sample of DNMT3A1 (long-isoform) and control in primed and early transition hESCs. e) qRT-PCR for DNMT3A and DNMT3B in control and knock-down naïve hESCs. Bars represent the mean of two biological replicates. Human GAPDH was used to normalise expression. f) qRT-PCR for DNMT3A/DNMT3B double knock down and control naïve hESCs. Bars represent the mean of three replicates and error bars represent the SEM. Human GAPDH was used to normalise expression. g) Raw protein intensity values for DNMT3A and DNMT3B extracted from proteomics data. Bars represent an average of 2 intensity values for each of the 3 replicates per sample, with error bars representing the SEM. Statistical difference between samples was analysed by a two-tailed student's t-test at each time point. ****$P<0.0001$. Source data are provided as a Source Data file.

Supplementary Figure 6: TET1 overexpression does not impact hypermethylation during resetting

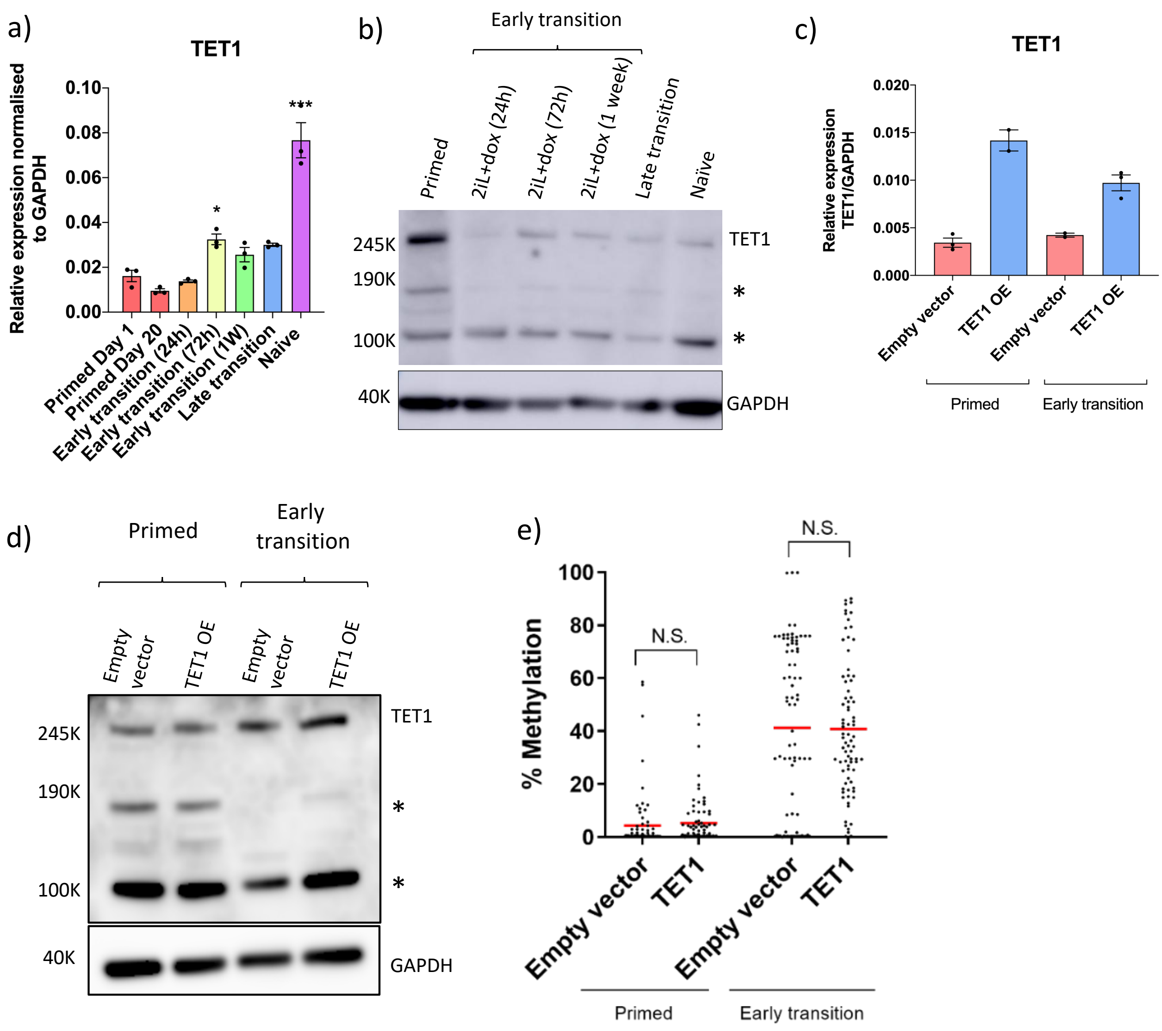

Supplementary Figure 6. TET1 overexpression does not impact hypermethylation during resetting. a) qRT-PCR for TET1 across the period of resetting. Bars are representative of the mean three biological replicates with two technical replicates. Error bars represent the standard error of the mean. Statistical difference between samples was analysed by a one-way ANOVA test. *P<0.05 and ***P<0.0001. Human GAPDH was used to normalise expression. b) Western blot analysis of TET1 across the transition from primed to naïve pluripotency, representative of 2 independent experiments. * denotes degraded fragments of TET1 protein. GAPDH is used as a loading control. c) qRT-PCR for TET1 in an empty vector control and overexpression cell line, in primed and early transition cells. Bars represent the mean of three technical replicates. Human GAPDH was used to normalise expression. d) Western blot analysis of TET1 in empty vector and TET1 overexpressing primed and early transition hESCs representative of 3 independent experiments. * denotes degraded fragments of TET1 protein. GAPDH is used as a loading control. e) Plot showing the % methylation in the early transition and primed state, for a TET1 overexpressing hESC line and empty vector control. Each dot represents the methylation % of single CpGs from 6 genomic regions analysed by targeted bisulfite sequencing, and the red bars represent the mean methylation level for each sample. Statistical difference between samples was analysed by a two-way ANOVA test, with Bonferroni post-hoc test of the TET1 overexpressing sample compared to the control (empty vector). N.S. denotes not significant (p > 0.05). Source data are provided as a Source Data file.

Supplementary Figure 7: SUSD2 can separate primed and naïve hESCs

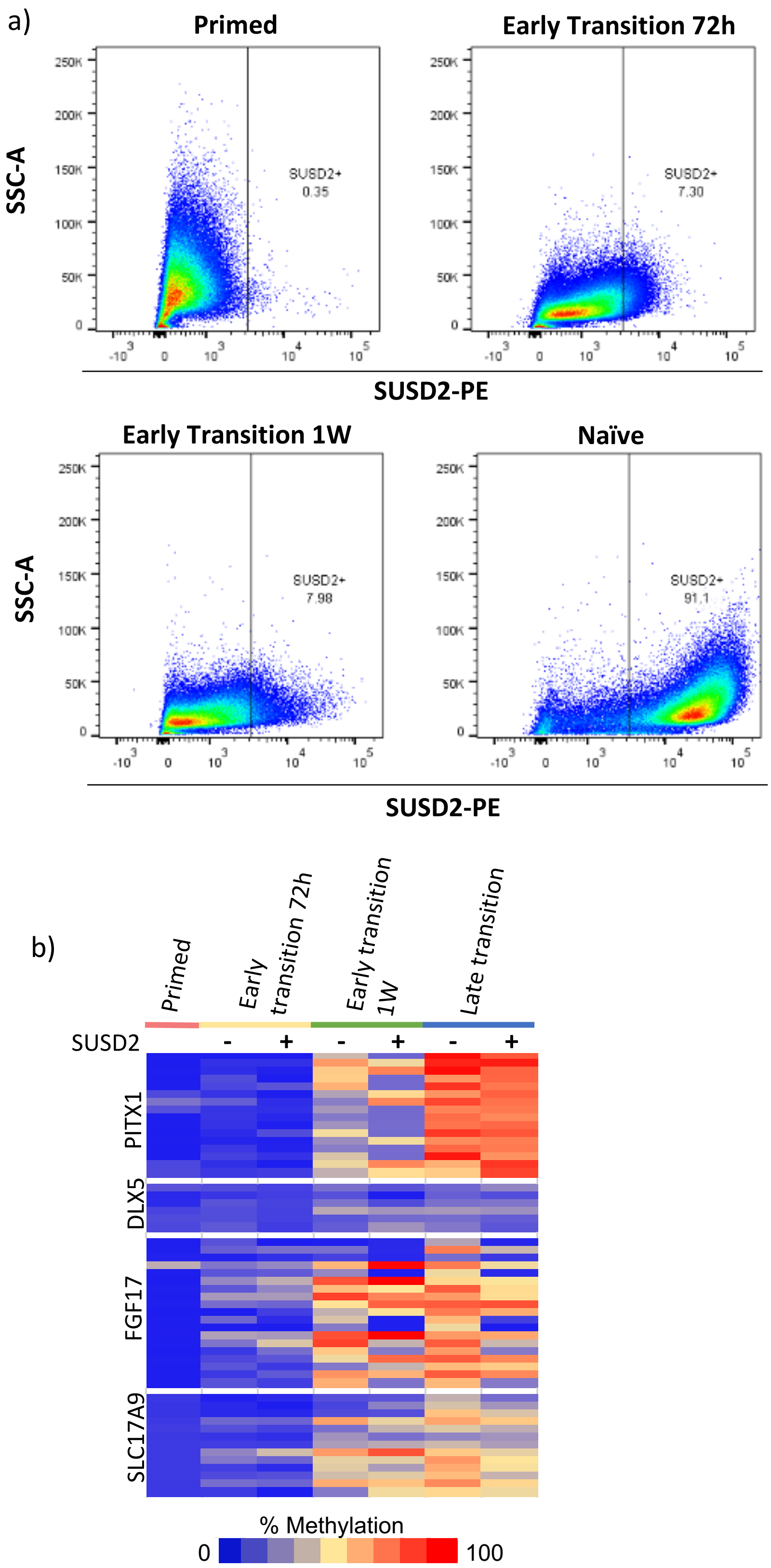

Supplementary Figure 7. **SUSD2 can separate primed and naïve hESCs.** a) Flow cytometry dot plots showing SUSD2-PE staining on the x-axis against the side scatter on the y-axis for hESCs across the time course of resetting. b) Targeted bisulfite-sequencing of 4 genomic regions. Each square represents the methylation % indicated by the colour key of a single CpG. The first column represents data from primed hESCs, and the subsequent columns represent data from early and late transition SUSD2+ and SUSD2- hESCs. Source data are provided as a Source Data file.

Supplementary Figure 8: Transcription factors knock downs

a)

qPCR of transcription factor knock down and control cell lines

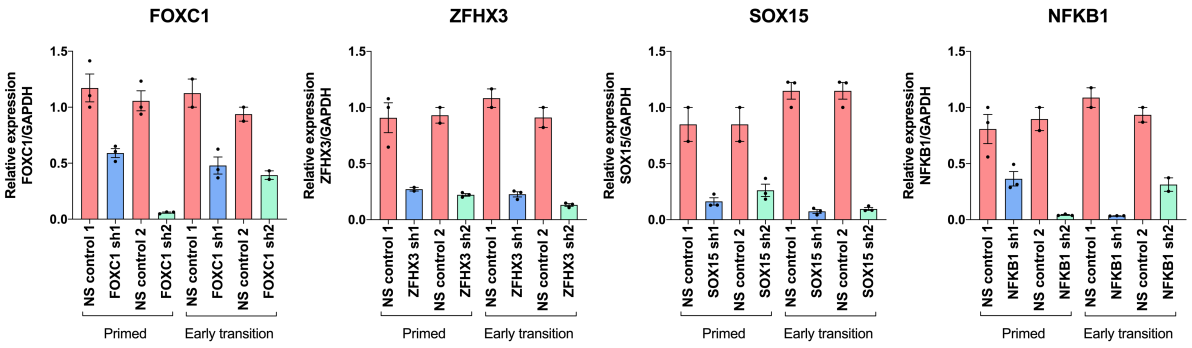

b)

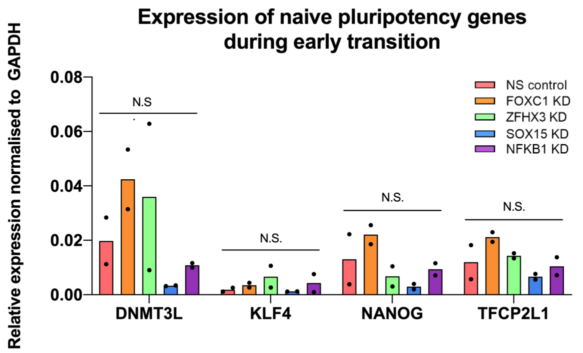

Supplementary Figure 8. Transcription factors knock downs. a) qRT-PCR for the transcripts of 4 transcription factors in control and knock-down cell lines, in primed and early transition cells. Bars represent the mean of three technical replicates and error bars represent the SEM. Human GAPDH was used to normalise expression. b) qRT-PCR for naïve pluripotency genes in control and transcription factor knock-down cell lines, in early transition hESCs. Bars represent the mean of three technical replicates, and 2 independent knock down cell lines. Error bars represent the standard error of the mean. Statistical difference between samples was analysed by a one-way ANOVA test. N.S. denotes not significant. Human GAPDH was used to normalise expression. Source data are provided as a Source Data file.

Supplementary Figure 9: Overlap of resetting-associated hypermethylation with cancer hypermethylation

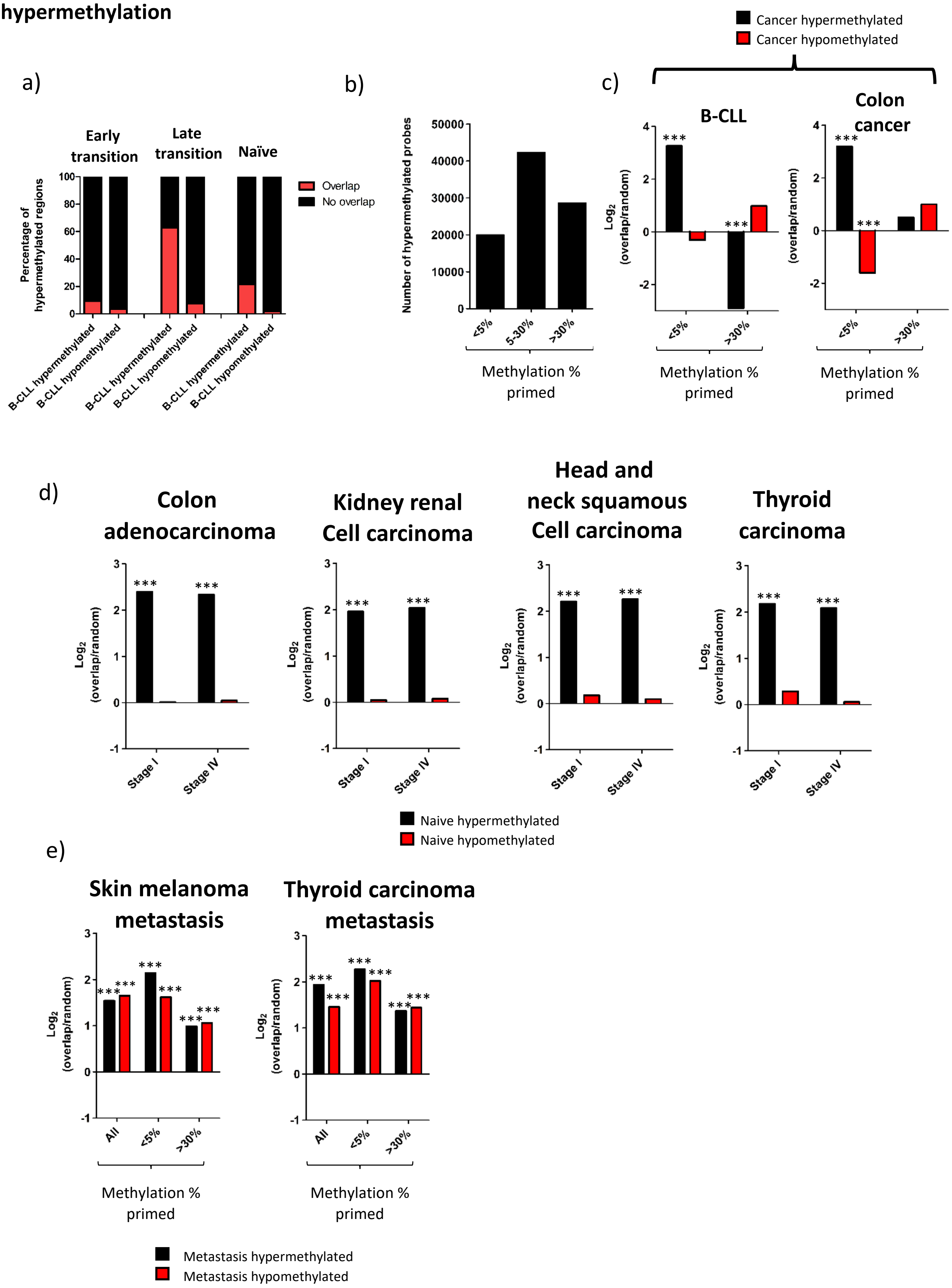

Supplementary Figure 9. Overlap of resetting-associated hypermethylation with cancer hypermethylation. a) Proportion of hypermethylated and hypomethylated regions in B-CLL (from published data) that overlap hypermethylated probes identified during each stage of hESC resetting (n = 46844 early transition, n = 91119 late transition, n = 20297 naïve). Data shows an enrichment for late transition hypermethylated probes within B-CLL hypermethylated regions (and to a lesser extent naïve hypermethylated probes). b) Bar plot showing the basal level of methylation in primed hESCs of probes that are hypermethylated during the late transition of resetting (n = 91119). c) Overlap of late transition hypermethylated probes (with <5% [n = 20064] or >30% [n = 28664] starting methylation in primed hESCs) with B-CLL and colon cancer hypermethylated regions from published data. d) Overlap of late transition DMPs (n = 91119 hypermethylated; n = 392,875 hypomethylated) with cancer hypermethylated probes obtained from TCGA. Overlaps were performed for both hyper- and hypomethylated probes compared to either stage I or stage IV hypermethylated probes (compared to normal controls) for each cancer type. e) Overlap of late transition hypermethylated and hypomethylated probes with probes differentially methylated in cancer metastasis (data from TCGA). Differential methylation for metastasis samples were generated via a pairwise comparison between primary cancer samples and cancer metastatic samples. Overlaps are shown for all late transition hypermethylation probes (as in 9d) as well as those that show low (<5%) and high (>30%) methylation in primed hESCs (as in 9c).
For overlap analysis, data is presented as the $\log_2$ corrected fold increase in the observed overlap compared to the mean overlap of 1000 randomly generated loci, where random loci generation was restricted to loci present in the Illumina EPIC array. For all figures ***P< 0.001.

Supplementary Figure 10: Resetting-associated hypermethylation across cancer types

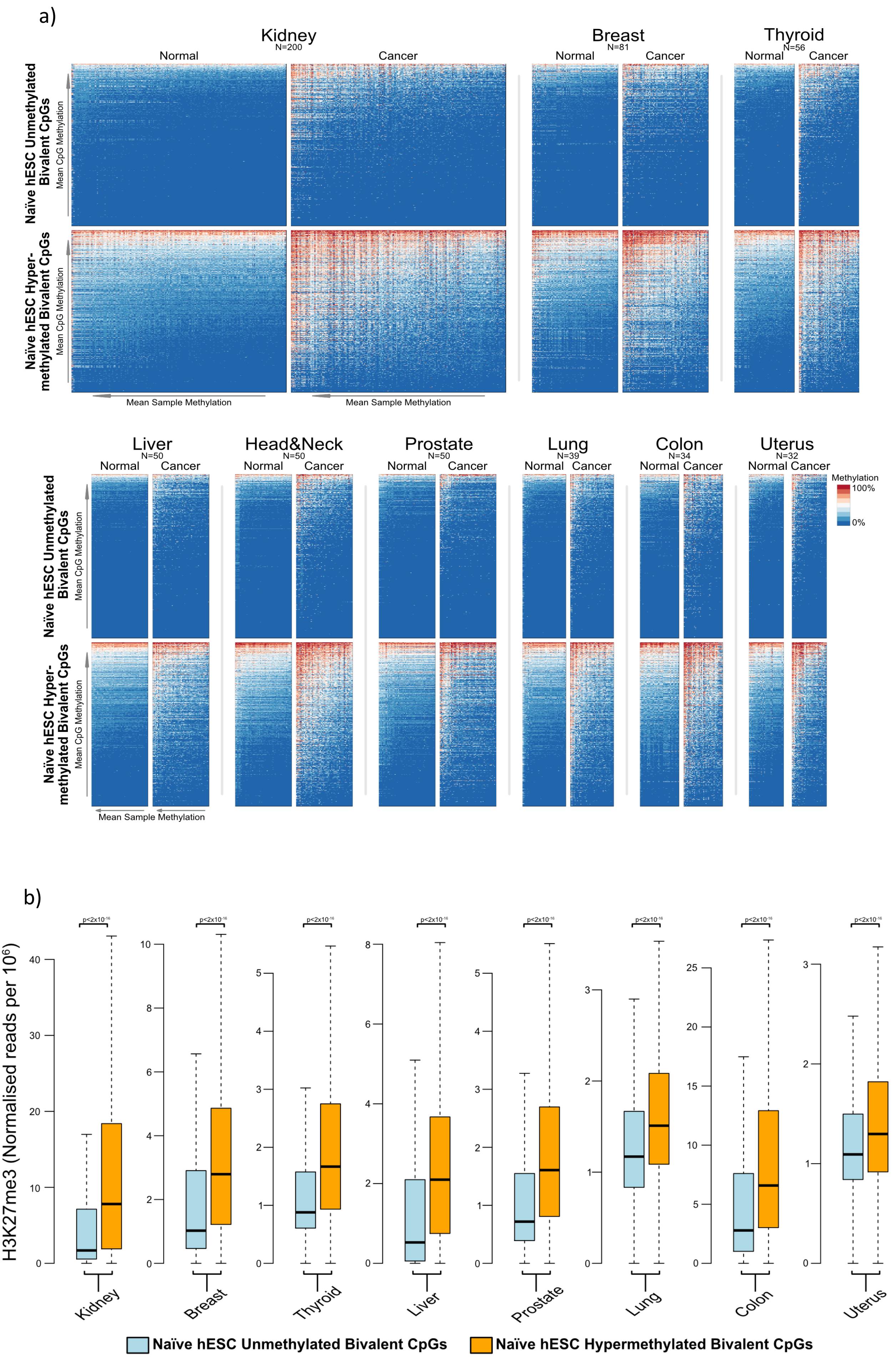

Supplementary Figure 10: Resetting-associated hypermethylation at H3K27me3 associated regions across cancer types. a) Heatmaps showing the methylation level of bivalent CpGs identified as hypermethylated (N=23123) or not hypermethylated (N=25977) in hESCs (as in Fig. 7a) during the transition from primed to naïve state, in tumour and corresponding normal tissue samples for a variety of cancer types, ordered by mean methylation level. Data was restricted to those cancer types that had at least 30 matched normal and cancer tissue datasets available. b) Boxplot of H3K27me3 reads within 250bp of the bivalent CpGs identified as hypermethylated (N=23123) or not hypermethylated (N=25977) in hESCs (as in Supplementary Fig. 10a). H3K27me3 ChIP data taken from ENCODE (see Supplementary Table 8) Lines=median; Box=25th–75th percentile; whiskers=1.5× interquartile range from box. P-values determined via two sample T-test (two-sided).

Supplementary Figure 11: Uncropped original Immunoblot scans and gating strategy for FACS/Flow Cytometry

Uncropped blot Supplementary Figure 6b

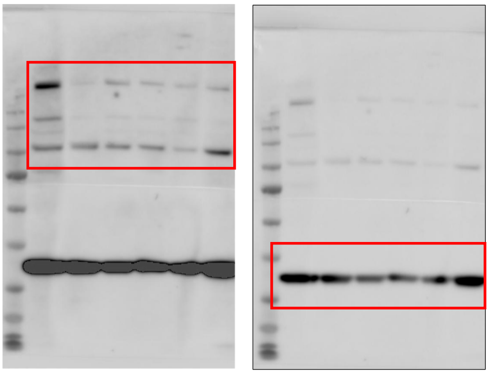

Uncropped blot Supplementary Figure 6d

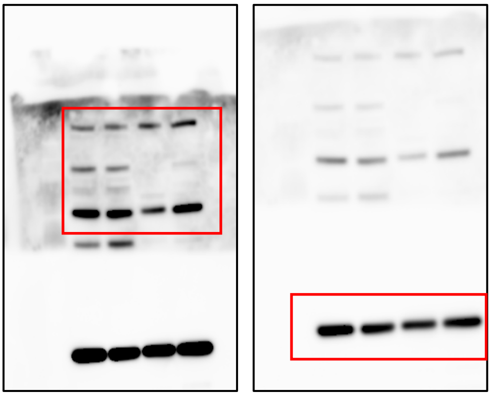

FACS/Flow cytometry gating strategy for Figure 4 and Supplementary Figure 7

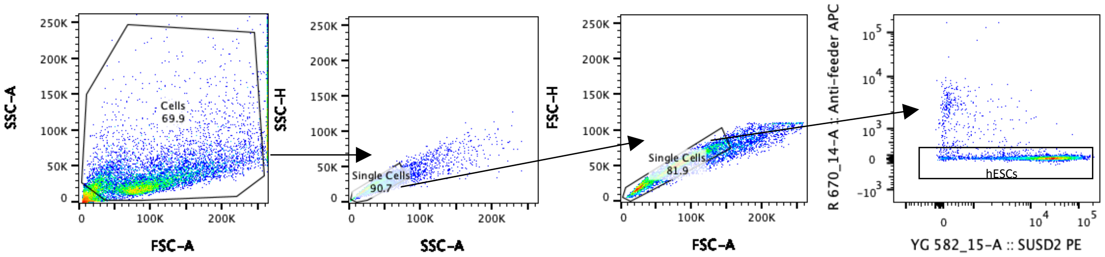

Supplementary Table 1: shRNA sequences (Dharmacon)

Target Gene	Mature antisense sequence	Identifier
DNMT3A	AATAATCTCCTTGACCTTGGG	TRCN0000035754
	TTATTAGCGAAGAACATCTGG	TRCN0000035757
DNMT3B	TATAGCAATTTGTCTTGAGGC	TRCN0000035684
	ATTTGAGAGATCGTTGCATGG	TRCN0000035686
DNMT3L	TTGTCCACGAACATCCAGAAG	TRCN0000019683
	TTCTTCTTCCGAAACCAGAGC	TRCN0000019680
DNMT3A iso1	ATTGGGTAATAGCTCTGAGGC	TRCN0000035756
FOXC1	AGGGTGATCTTCTTGTCCGGG	TRCN0000013964
	TTCAGGTACCACGAGGTGAGG	TRCN0000013965
ZFHX3	ATTCTCATAATTCTTCCTGGC	TRCN0000013558
	TTAGCTGTGGAAACTAAAGGG	TRCN0000013560
SOX15	TTCCAGTTTGCAGTGGGAAGA	TRCN0000019150
	TAGTGGGTATAGGTGGGCAGC	TRCN0000019151
NFKB1	ATCATCAGATGTAAACTCTGG	TRCN0000006518
	TTCAGGATAGTAGAGGAAAGG	TRCN0000006520

Supplementary Table 2: Primer Sequences used for qPCR

Gene	Primer Sequence (5' – 3')
GAPDH	GATTTGGTCGTATTGGGCGC
	TTCCCGTTCTCAGCCTTGAC
DNMT3A	AGTACGACGACGACGGCTA
	CACACTCCACGCAAAAGCAC
DNMT3B	GAGTCTGCACGGGACCTATT
	GTCACGGGGAGGGATTTAGC
DNMT3L	CATAGCCTGGTGACCTCTGG
	CTGGTGGGTTCAAGGTTCCA
DNMT3A iso1	ACTACATCAGCAAGCGCAAG
	CACAGCATTCATTCCTGCAA
DNMT3A iso2	GCTGCACCTGGCCTTATG
	CGTCTTTCAGGCTACGATCC
TET1	CGAGTTGGAAAGTTTGCCCG
	CACAAGGTTTTGGTCGCTGG
FOXC1	GCTGTCAAATGGCCTTCCCT
	TCCTGCTTTGGGGTTCGATT
ZFHX3	CTTCCAGAGGAGGACGAGGA
	AATGGCTTCTTCTGGGTCCG
SOX15	GGCTTTGGGTACAGACCCC
	GTTTGCAGTGGGAAGAGCCAT
NFKB1	GCTTAGGAGGGAGAGCCCA
	CTGCCATTCTGAAGCCGGG

Supplementary Table 3: Primer sequences used for ChIP-qPCR

Gene	Primer Sequence (5' – 3')
COL11A1	AGAGAACTGCACGTCCAACC
	TGCAACCAAGTGAGAAGCAG
DLX5	GGCAATCTGGGAGTTCCACA
	CTGAGCGGGGCTGTATCTTG
TRPC4	TATATGCACCCAGATGCCCC
	TTTAAAGCAGGGGAGAGGGC
PITX1	CATACACAGGGACGCTGTAAAC
	GGGAGGTCCATCTCAGAACA
SIM1	GCCTGGGGAGTAAGGAGACT
	AACTTCCTTCCGCTGGTAGC
SLC17A9	GCTCCTATGGGGCAGCAG
	GGCTTGGGGTGCTCAGAC
LIN9	ATCTCAGGCACGTTGGTTTC
	CCAGTGACTCACCCAATCCT
WTIP	GGTTGGGACGAGGAAGGT
	AGTGTGCCCATGAACCTGAC
DPP6	TATTGGTAGCGGCCAAAAAG
	GATCATGGCCTTTTCCTTCA

Supplementary Table 4: Primer Sequences used for targeted bisulfite sequencing

Gene/region	Primer Sequence (5' – 3')
DLX5	TGTTTAGTATTAGTTTAGTTTTATTTGGAGTGTGG
	AATCCAAACCRCAAAAACAAAAAATTAATACAC
PITX1	GGGGTTGTTYGTTTAGATAGAGGGTTATTTTTTAG
	TTAAAAAAACRATACCCCCAACCCAAAATC
FGF17	GGTAYGAGGGTTGGTTTATGG
	AATAAAAAAACRACCCAAAAACTACTACCCCCTAC
SLC17A9	TTGGTTTYGTTTTTATGGGGTAGTAGGG
	TATATCCCRAAACTACCTCCCAACCCAAACTAACC

Supplementary Table 5: Primer sequences for Fluidigm targeted bisulfite sequencing

Gene/region	Primer Sequence (5' – 3')
DLX5	TGTTTAGTATTAGTTTAGTTTTATTTGGAGTGTGG
	AATCCAAACCRCAAAAACAAAAAATTAATACAC
ZFHX3	GAGATGTTGATTTAGAGTTTTTTTT
	ACCACCTAAAATCCCTCTACTTCTT
FBXL13	GTAATTGGGGTTAGTTGGATGTTAG
	AAACAACACATAAACTAATTTTCTTTCTTA
RAB34	AGGTTTGGGAGGTGATTTATAGAGT
	ACAATAAACACCCATACCAAAAAAA
ACHE	GAAGGAAGGGAAGGTTTAGTTTAGA
	TTTAAAAAAATCTCAAAACATCCTAAC
SIM1	TTTTTTGAGAGAGTGTAGGAGAGTTT
	ACTAATTACACCAATTTCCCTCTCTT
TBX4	GGGTTTTAGATATAGTTGGATTTAG
	ACCCATAAAAATAAAATTAACAAAC
NFIX	TAGTAAATTGAAAGGATTAGTGAAT
	TCTAACCCCTACAAAAAATAACACC
NR2F2	TTATTAATTGTGGAGTGTTTTTTTT
	ATACCCATAATATTATTAAACTACATACAT
SHH	ATAGTAGGTTTGATAGAGATTTGGG
	ACTACAAATAACAACTCACCTAACC
TBX5	AAAGTAAAGATTTTTAAGGTTGGTT
	TTCCTATTCCCCCAAAAAAAA
FGF17	GGTAYGAGGGTTGGTTTATGG
	AATAAAAAAACRACCCAAAAACTACTACCCCCTAC
NKX6-1	TTGATTTGTGAGAATTAATAAATAA
	ACAATAAACTCCCTAACTATTTAAC
PITX1	GGGGTTGTTYGTTTAGATAGAGGGTTATTTTTTAG
	TTAAAAAAACRATACCCCCAACCCAAAATC
ITGAM	TGGGGAATTTTTAGAAATTTAGAGT
	CCCCAATCACACAACTAACAAC
PAX8	TTAATTTTTGGGTGATATATTTGGT
	ATTTCTAACTCCTAAATCCACTCAAC
SLC17A9	TTGGTTTYGTTTTTATGGGGTAGTAGGG
	TATATCCCRAAACTACCTCCCAACCCAAACTAACC

Supplementary Table 6: Primer sequences used for glucMS-qPCR

CpG	Primer Sequence (5' – 3')
HoxD-CpG1	AGCTCAAACCCAAGCCAATA
	TCTGGGTAAGTCGGAGGAAG
HoxD-CpG2	CTTCTCGCAGCCCTACCT
	TTCGGGCGCATAGAACTTA
HoxD-CpG3	AGTTCTATGCGCCCGAAG
	GCTTTGAGAGCAGCCACTG
