# Supplementary material for: Transition to naïve human pluripotency mirrors pan-cancer DNA hypermethylation
Source: Nat Commun. 2020 Jul 22;11:3671. doi: 10.1038/s41467-020-17269-3 (PMC7376100; doi:10.1038/s41467-020-17269-3)
Supplement: Supplementary file 3 — Description of Additional Supplementary Files [file 41467_2020_17269_MOESM3_ESM.docx]

Description of Additional Supplementary Files

Title: Supplementary Data 1

Description: Analysis of H3K27me3 in Human Tissue

Title: Supplementary Data 2

Description: Proteomics analysis of hESC resetting
