# Supplementary material for: Transition to naïve human pluripotency mirrors pan-cancer DNA hypermethylation
Source: Nat Commun. 2020 Jul 22;11:3671. doi: 10.1038/s41467-020-17269-3 (PMC7376100; doi:10.1038/s41467-020-17269-3)
Supplement: Supplementary file 6 — Reporting Summary [file 41467_2020_17269_MOESM6_ESM.pdf]

nature research

Corresponding author(s): Gabriella Ficz

Last updated by author(s): Jun 10, 2020

# Reporting Summary

Nature Research wishes to improve the reproducibility of the work that we publish. This form provides structure for consistency and transparency in reporting. For further information on Nature Research policies, see Authors & Referees and the Editorial Policy Checklist .

## Statistics

For all statistical analyses, confirm that the following items are present in the figure legend, table legend, main text, or Methods section.

| n/a | Confirmed | |
|---|---|---|
| ☐ | ☒ | The exact sample size (*n*) for each experimental group/condition, given as a discrete number and unit of measurement |
| ☐ | ☒ | A statement on whether measurements were taken from distinct samples or whether the same sample was measured repeatedly |
| ☐ | ☒ | The statistical test(s) used AND whether they are one- or two-sided<br>*Only common tests should be described solely by name; describe more complex techniques in the Methods section.* |
| ☒ | ☐ | A description of all covariates tested |
| ☐ | ☒ | A description of any assumptions or corrections, such as tests of normality and adjustment for multiple comparisons |
| ☐ | ☒ | A full description of the statistical parameters including central tendency (e.g. means) or other basic estimates (e.g. regression coefficient) AND variation (e.g. standard deviation) or associated estimates of uncertainty (e.g. confidence intervals) |
| ☐ | ☒ | For null hypothesis testing, the test statistic (e.g. *F*, *t*, *r*) with confidence intervals, effect sizes, degrees of freedom and *P* value noted<br>*Give P values as exact values whenever suitable.* |
| ☒ | ☐ | For Bayesian analysis, information on the choice of priors and Markov chain Monte Carlo settings |
| ☒ | ☐ | For hierarchical and complex designs, identification of the appropriate level for tests and full reporting of outcomes |
| ☒ | ☐ | Estimates of effect sizes (e.g. Cohen's *d*, Pearson's *r*), indicating how they were calculated |

*Our web collection on statistics for biologists contains articles on many of the points above.*

## Software and code

Policy information about availability of computer code

| | |
|---|---|
| Data collection | No custom software was used.<br>The following publicly available software was used for analysis:<br>Seqmonk (v1.41.0), AME on the MEME suite (v5.0.4), regioneR (v3.8), Bisulfite primer seeker (Zymo), Methprimer (Urogene) Bismark (v.0.19.0), ChAMP (v2.11.3), Mascot Daemon (v2.5.0), Mascot Distiller (v2.5.1), Mascot Search Engine (v2.5), hisat2 (v2.1.0), FeatureCounts (Subread, v1.6.3), EdgeR (v3.18.1), FlowJo (v10) and Prism (v7.04, v.8.4.2) |
| Data analysis | Data was analyzed using standard computational pipelines that are highlighted in Methods when used. |

For manuscripts utilizing custom algorithms or software that are central to the research but not yet described in published literature, software must be made available to editors/reviewers. We strongly encourage code deposition in a community repository (e.g. GitHub). See the Nature Research guidelines for submitting code & software for further information.

## Data

Policy information about availability of data

All manuscripts must include a data availability statement. This statement should provide the following information, where applicable:

- Accession codes, unique identifiers, or web links for publicly available datasets
- A list of figures that have associated raw data
- A description of any restrictions on data availability

All datasets have been deposited in the Gene Expression Omnibus and will be accessible under GSE128130 upon publication (currently is scheduled to be released on Jun 12, 2020). Additional data used include ENCODE, ChromHMM and TCGA pan-cancer data, HOCOMOCO (v11 FULL), SwissProt (Dec 2015 release). Data for human naive reprogramming methods was downloaded from GSE60945, GSE76970, GSE90168, and data for human and mouse in vivo development from GSE34864 and GSE49828. No restrictions on data availability.

# Field-specific reporting

Please select the one below that is the best fit for your research. If you are not sure, read the appropriate sections before making your selection.

☒ Life sciences ☐ Behavioural & social sciences ☐ Ecological, evolutionary & environmental sciences

For a reference copy of the document with all sections, see nature.com/documents/nr-reporting-summary-flat.pdf

# Life sciences study design

All studies must disclose on these points even when the disclosure is negative.

| | |
|---|---|
| Sample size | For experimental work, we used three biological replicates as indicated in the figure legends and methods, unless stated otherwise.<br>For comparisons to TCGA pan-cancer data, we chose all cancer types that n > 30 patient matched samples. i.e. over 30 individuals that had had both normal tissue and a tumour sample of that type profiled.<br>A sample size calculation was not performed, but a minimum of 3 biological replicates was performed where possible, to allow for an indication of both experimental uncertainties and biological diversity to inform the validity of our hypothesis testing. |
| Data exclusions | No samples were excluded from analysis. |
| Replication | Replicates and results are reported and clearly described in the Figures, Legends and Methods. In general, at least 3 replicates were undertaken per measurement (technical and wherever possible >2 biological replicates), however on rare occasions one of these replicates failed to provide any value (for example, a qPCR replicate sample that fails to amplify). In all cases, all replicates that provided any value are included in the analysis (i.e. no outliers are excluded), and graphs have points marked for each replicate where appropriate to assist in viewing exactly how many replicates were used, and with what variability in outcomes. |
| Randomization | Randomization was not applicable to this study. Bias was not a significant risk of the experimental design as internal controls were used where appropriate. |
| Blinding | Blinding was not applicable to this study as human participants were not used in the research. |

# Reporting for specific materials, systems and methods

We require information from authors about some types of materials, experimental systems and methods used in many studies. Here, indicate whether each material, system or method listed is relevant to your study. If you are not sure if a list item applies to your research, read the appropriate section before selecting a response.

## Materials & experimental systems

| n/a | Involved in the study | |
|---|---|---|
| ☐ | ☒ | Antibodies |
| ☐ | ☒ | Eukaryotic cell lines |
| ☒ | ☐ | Palaeontology |
| ☒ | ☐ | Animals and other organisms |
| ☒ | ☐ | Human research participants |
| ☒ | ☐ | Clinical data |

## Methods

| n/a | Involved in the study | |
|---|---|---|
| ☒ | ☐ | ChIP-seq |
| ☐ | ☒ | Flow cytometry |
| ☒ | ☐ | MRI-based neuroimaging |

## Antibodies

| | |
|---|---|
| Antibodies used | 1) Rabbit polyclonal anti-TET1 [N3C1] - Source Bioscience, Cat #GTX124207<br>2) Monoclonal rabbit anti-GAPDH [14C10] - Cell Signaling Technologies, Cat#2118S<br>3) Rabbit polyclonal anti-H3K27me3 - Abcam, Cat#ab195477<br>4) Rabbit polyclonal anti-H3K4me3 - Abcam, Cat #ab8580<br>5) Mouse monoclonal anti-SUSD2-PE - Biolegend, Cat#327406<br>6) Mouse anti-feeder-APC - Miltenyi Biotec, Cat#130-120-802<br>7) Mouse anti-feeder-PE - Miltenyi Biotec, Cat130-120-166<br>8) Amersham ECL Mouse IgG, HRP-linked whole Ab - GE healthcare, Cat#NA931<br>9) Amersham ECL Rabbit IgG, HRP-linked whole Ab - GE healthcare, Cat #NA934 |
| Validation | All antibodies used in this study have been commercially validated and are widely used by the scientific community. The less frequently used TET1 antibody has been validated in KO/KD and overexpression systems and referenced in many publications. The SUSD2 antibody is the same that was used in previously in the human naive stem cells by Austin Smith's lab (PMID: 31031191). All other antibodies are part of well established kits and have been used extensively worldwide. |

# Eukaryotic cell lines

Policy information about cell lines

| | |
|---|---|
| Cell line source(s) | H9-NK2 human embryonic stem cells were generated and provided by Prof. Austin Smith at the WT–MRC Cambridge Stem Cell Institute, with permission from Wi-Cell. |
| Authentication | H9-NK2 cell line was not authenticated but throughout the study we tracked markers known to change during resetting, which was reproducible and stable over many years. Where possible we stored and used early passage populations to further validate our findings. |
| Mycoplasma contamination | Negative for mycoplasma contamination. |
| Commonly misidentified lines (See ICLAC register) | No commonly misidentified cell lines were used. |

# Flow Cytometry

## Plots

Confirm that:

- [x] The axis labels state the marker and fluorochrome used (e.g. CD4-FITC).
- [x] The axis scales are clearly visible. Include numbers along axes only for bottom left plot of group (a 'group' is an analysis of identical markers).
- [x] All plots are contour plots with outliers or pseudocolor plots.
- [x] A numerical value for number of cells or percentage (with statistics) is provided.

## Methodology

| | |
|---|---|
| Sample preparation | Human embryonic stem cells were dissociated to single cells with Accutase and washed with 3%FCS/PBS, before being blocked in 10% FCS/PBS. Cells were resuspended in 2.5ul SUSD2-PE antibody (Biolegend; 327406) and 5ul anti-feeder-APC antibody (Miltenyi Biotec; 130-120-802) for 15 minutes at 4°C in the dark. Alternatively, following 1 hour pulse labelling with 10um BrdU, cells were fixed, permeabilised, blocked and stained using the APC BrdU Flow Kit (BD Pharminogen) following manufacturer's instructions, with the addition of 5ul of anti-feeder-PE antibody (Miltenyi Biotec; 130-120-166). Cells were wash twice in 3%FCS/PBS and then stained with DAPI for 15 minutes at 4°C in the dark. |
| Instrument | Samples were either analysed on an LSR Fortessa cell analyser (BD Biosciences) or FACS sorted on the BD FACS Aria Fusion cell sorter. |
| Software | Flow cytometry data analysis was carried out using FlowJo Version 10 software. |
| Cell population abundance | Sorted samples were used directly for DNA isolation. |
| Gating strategy | Cells were gated based on the FSC-A and SSC-A and singlets were selected based on SSC-A and SSC-H, and further by FSC-A and FSC-H. Following this, fluorophores of interest were gated against SSC-A using an unstained sample as a negative population. |

- [x] Tick this box to confirm that a figure exemplifying the gating strategy is provided in the Supplementary Information.
